# ORCA-EFCD consensus report on clinical recommendation for caries diagnosis. Paper I: caries lesion detection and depth assessment

**DOI:** 10.1007/s00784-024-05597-3

**Published:** 2024-03-22

**Authors:** Jan Kühnisch, Johan KM Aps, Christian Splieth, Adrian Lussi, Anahita Jablonski-Momeni, Fausto M. Mendes, Gottfried Schmalz, Margherita Fontana, Avijit Banerjee, David Ricketts, Falk Schwendicke, Gail Douglas, Guglielmo Campus, Monique van der Veen, Niek Opdam, Sophie Doméjean, Stefania Martignon, Klaus W. Neuhaus, Keith Horner, Marie-Charlotte DNJM Huysmans

**Affiliations:** 1 Department of Conservative Dentistry and Periodontology, University Hospital, Ludwig-Maximilians Universität München, Poliklinik für Zahnerhaltung und Parodontologie, Goethestraße 70, 80336 München, Germany; 2 OpiniDent BV, Kortrijk, Belgium; 3 Preventive and Pediatric Dentistry, Center for Oral Health, Universitätsmedizin Greifswald, Greifswald, Germany; 4 University Hospital for Conservative Dentistry and Periodontology, Medical University of Innsbruck, Innsbruck, Austria; 5 Department of Restorative, Preventive and Pediatric Dentistry, School of Dental Medicine, University of Bern, Bern, Switzerland; 6 Department of Orthodontics, Dental School, Philipps-University Marburg, Marburg, Germany; 7 Department of Pediatric Dentistry, School of Dentistry, University of São Paulo, São Paulo, Brazil; 8 Department of Conservative Dentistry and Periodontology, University Hospital Regensburg, Regensburg, Germany; 9 Department of Periodontology, University of Bern, Bern, Switzerland; 10 Department of Cariology, Restorative Sciences and Endodontics, University of Michigan School of Dentistry, Ann Arbor, USA; 11 Conservative & MI Dentistry, Faculty of Dentistry, Oral & Craniofacial Sciences, King’s College London, London, UK; 12 Unit of Restorative Dentistry, University of Dundee, Dundee, UK; 13 Department of Dental Public Health, University of Leeds Dental School, Leeds, UK; 14 Department of Surgery, Microsurgery and Medicine Sciences, School of Dentistry, University of Sassari, Sassari, Italy; 15 Departments of Preventive Dentistry and Paediatric Dentistry, Academic Centre for Dentistry Amsterdam, University of Amsterdam and VU University, Amsterdam, The Netherlands; 16 Oral Hygiene School, Inholland University of applied sciences, Amsterdam, The Netherlands; 17 Department of Dentistry, Radboud University Medical Center, Nijmegen, The Netherlands; 18 Centre de Recherche en Odontologie Clinique EA 4847, UFR d’Odontologie, Département d’Odontologie Conservatrice, Université Clermont Auvergne, Clermont-Ferrand, France; 19 Service d’Odontologie, CHU Estaing Clermont-Ferrand, Clermont-Ferrand, France; 20 UNICA - Caries Research Unit, Research Department, Universidad El Bosque, Bogotá, Colombia; 21 Department of Pediatric Oral Health, University Center for Dental Medicine Basel (UZB), University of Basel, Basel, Switzerland; 22 Department of Dermatology, Inselspital, Bern University Hospital, University of Bern, Bern, Switzerland; 23 Division of Dentistry, School of Medical Sciences, Faculty of Biology, Medicine and Health, University of Manchester, Manchester Academic Health Science Centre, Manchester, UK

**Keywords:** Caries detection, Diagnosis, Assessment, Visual examination, Dental radiography, Bitewing radiography, Adjunct methods, Laser fluorescence

## Abstract

**Objectives:**

The aim of the present consensus paper was to provide recommendations for clinical practice considering the use of visual examination, dental radiography and adjunct methods for primary caries detection.

**Materials and methods:**

The executive councils of the European Organisation for Caries Research (ORCA) and the European Federation of Conservative Dentistry (EFCD) nominated ten experts each to join the expert panel. The steering committee formed three work groups that were asked to provide recommendations on (1) caries detection and diagnostic methods, (2) caries activity assessment and (3) forming individualised caries diagnoses. The experts responsible for “caries detection and diagnostic methods” searched and evaluated the relevant literature, drafted this manuscript and made provisional consensus recommendations. These recommendations were discussed and refined during the structured process in the whole work group. Finally, the agreement for each recommendation was determined using an anonymous Delphi survey.

**Results:**

Recommendations (*N* = 8) were approved and agreed upon by the whole expert panel: visual examination (*N* = 3), dental radiography (*N* = 3) and additional diagnostic methods (*N* = 2). While the quality of evidence was found to be heterogeneous, all recommendations were agreed upon by the expert panel.

**Conclusion:**

Visual examination is recommended as the first-choice method for the detection and assessment of caries lesions on accessible surfaces. Intraoral radiography, preferably bitewing, is recommended as an additional method. Adjunct, non-ionising radiation methods might also be useful in certain clinical situations.

**Clinical relevance:**

The expert panel merged evidence from the scientific literature with practical considerations and provided recommendations for their use in daily dental practice.

## Introduction

**Fig. 1:**
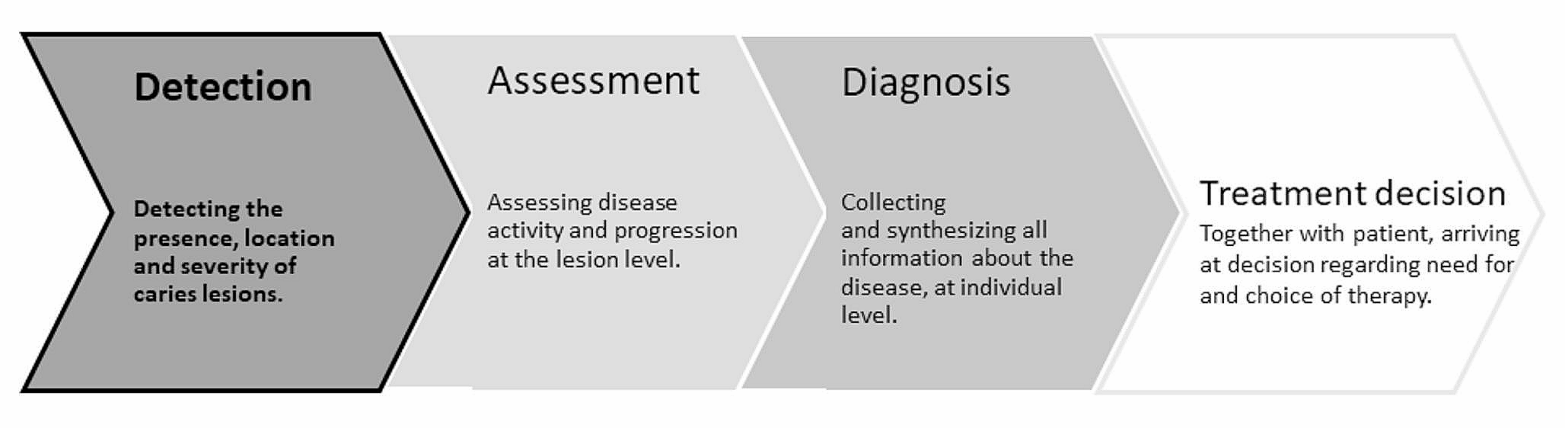
Steps in the caries diagnostic process and selection of therapy

Far too often, caries diagnosis is limited to the determination of the presence or absence of cavitation. In contrast, early detection provides the opportunity to arrest caries or to treat lesions by treating lesions with non-operative or minimally invasive techniques. Therefore, appropriate caries management needs to be based upon diagnostic methods and indices that cover all severity stages of caries as well as their activity status (Fig. 1). Many methods have been developed, evaluated and proposed for the clinical detection of caries on occlusal, proximal, smooth or root surfaces. When considering their practical relevance, it must be stated that only visual examination (VE), visual-tactile examination and radiographic examination have gained worldwide acceptance. Adjunct methods, e.g., laser fluorescence, fluorescence imaging or (near-)infrared light (trans)illumination (NILT), are not commonly used, although they provide additional information using ionising radiation-free technologies. In addition to the usability and availability of any diagnostic method, its validity in terms of accuracy is of clinical importance. Furthermore, the reproducibility of measurements must be considered another key variable. The aim of this ORCA-EFCD consensus paper was to provide expert recommendations for clinical practice considering the use of VE, dental radiography and adjunct methods for primary caries detection in all patient groups. The expert group merged existing evidence from the scientific literature with practical considerations and provided recommendations for their use in daily dental clinical practice. In addition to this paper, clinical recommendations for caries activity assessment and the individual diagnosis of caries were developed in parallel projects [1, 2].

## Materials and methods

*Expert panel.* The executive councils of the European Organisation for Caries Research (ORCA) and the European Federation of Conservative Dentistry (EFCD) agreed in 2021 to develop consensus statements on (1) caries detection and diagnostic methods, (2) caries activity/progression assessment and (3) individualised caries diagnosis. The previously jointly published statements on caries management [3–10] served as a model for this process. Both academic societies identified and nominated ten experts each to join the development process. MCD Huysmans, C. Splieth, K. Neuhaus and J. Kühnisch formed the steering committee for the project. In detail, J. Kühnisch moderated the process of caries detection and diagnostic methods, and the other work group members were Johan K. M. Aps, Keith Horner, Anahita Jablonski-Momeni, Adrian Lussi, Fausto M. Mendes and Gottfried Schmalz.

*Search of the literature.* The basis for the consensus process was a search of literature. PubMed and Google Scholar were searched for articles published from January 1, 2010, to June 31, 2022, using a search term for systematic reviews, meta-analysis and/or guidelines on caries diagnostic/detection methods (Table 1). The identified papers were screened systematically for relevance by the work group.

**Table 1:** Search string used for the identification of potential publications in PubMed

Search string	
((Dental Caries OR caries) AND (detect* OR diagn* OR assessment* OR evaluat*) AND (visual OR radiograph* OR bitewing OR adjunct OR additional OR light) AND (guideline OR guidance OR recommendation* OR “practice guidelines” OR consensus OR “systematic review” OR “meta-analysis”)) 2012–2022	

*Structured development of the recommendations in the expert group “Caries detection methods*”. It was considered that a group consensus process is crucial in developing clinical recommendations [11]. The approach for the recommendations in this paper included a systematic search of the literature, multiple online meetings and a structured communication flow aiming to converge existing opinions and, finally, efforts to reach a unanimous group consensus. The experts identified practical needs from different perspectives, e.g., patient needs, potential and limitations of national health care systems, recent developments in caries epidemiology, availability and acceptance of diagnostic methods among dental practitioners, and drafted a working paper including prephrased recommendations for discussion by the whole expert panel, completed in July 2022.

*Level of evidence.* Furthermore, the level of evidence from the available literature was evaluated by the working group in accordance with published recommendations. Evidence supported by unequivocal scientific studies, e.g., multiple randomised controlled clinical trials or systematic reviews/meta-analyses, was evaluated as “High”. Evidence based on well-designed clinical studies, e.g., controlled clinical trials, was evaluated as “moderate”. Finally, evidence based on expert opinion only or that are based on weak evidence, e.g., laboratory (*in vitro)* studies or only low-quality studies or studies with contradicting results were ranked as “Low”.

*Level of agreement.* The structured consensus process was initiated during an online group workshop on August 31, 2022. During this meeting, the existing scientific literature and empirical experiences were presented to the whole expert group and critically discussed, and the recommendations arising from this work were reviewed and rephrased until consensus was reached. Some nominees were unable to attend the meeting, but they had full access to all documents and were invited to suggest changes in the main text and recommendations. The present manuscript was finalised by the work group on the basis of the discussions at the meeting. After this, all recommendations were redistributed to the expert panel, giving all experts the opportunity for critical rereading. Subsequently, all recommendations and documents were harmonised. The final manuscript versions were reviewed again by the whole expert panel before submission for publication.

Finally, a confidential web-based Delphi survey (Castor EDC, Amsterdam, the Netherlands) was undertaken as carried out in earlier ORCA-EFCD consensus papers [3–10]. During the voting process, all experts gradually agreed independently of each other on every single recommendation. It was possible to vote from grade 1 (completely disagree) to grade 10 (completely agree). An additional field for free-text comments was made available to allow reasoning for a certain decision or proposals for modifications. The level of agreement was calculated for each item as the median value out of all votings. At least 80% of the vote ≥ 8 was considered as acceptance of the statement by the group, and the results were reported as agreement (10 − 8), (neutral 7 − 4) or disagreement (3 − 1).

### Visual examination

**Fig. 2:**
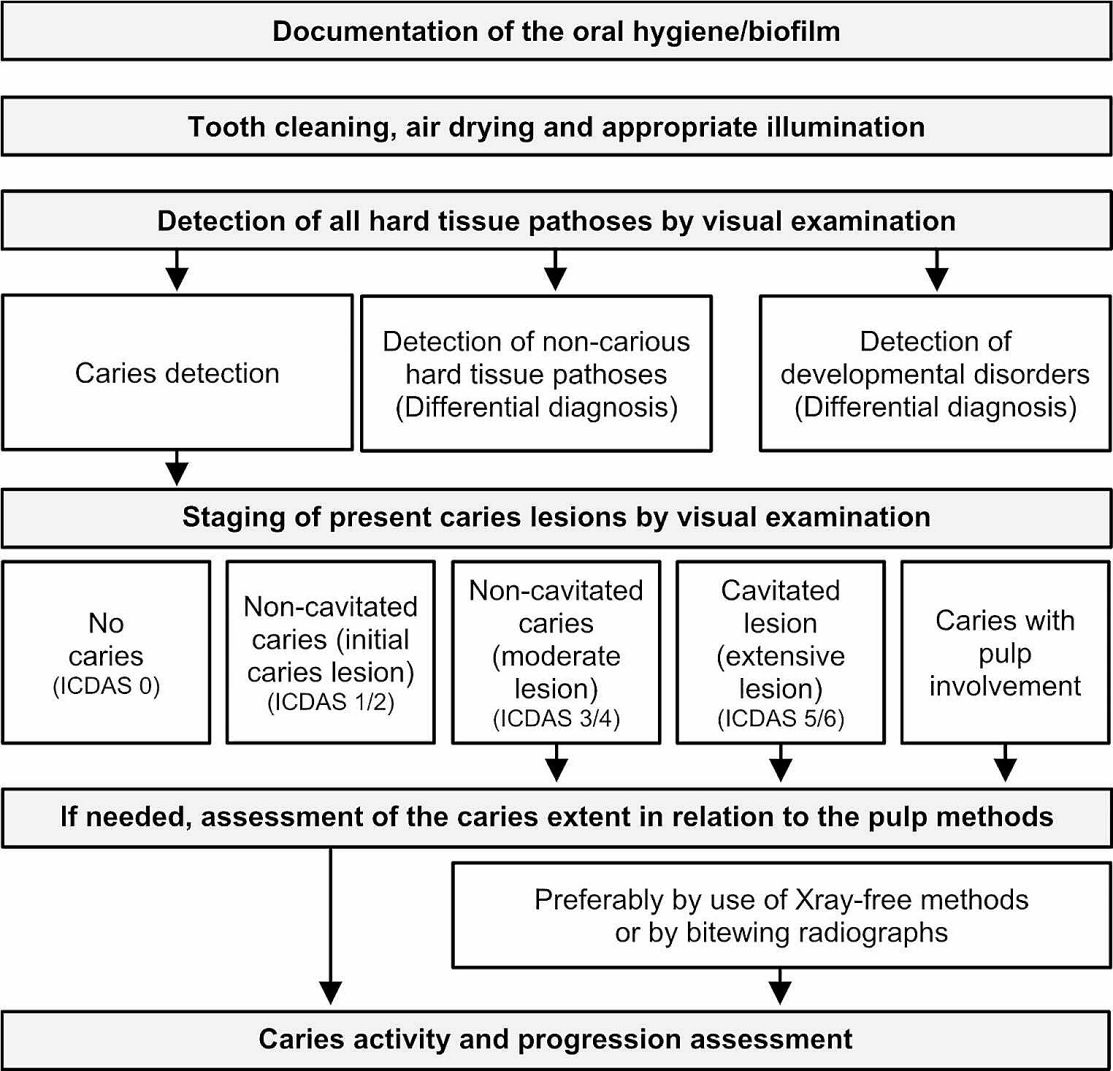
Scheme for a structured diagnostic examination

Several visual scoring systems or indices for the assessment of dental caries have been developed and published, including the International Caries Detection and Assessment System (ICDAS) [12–18] and its earlier proposals [19, 20], the Universal Visual Scoring System (UniViSS) [21, 22] and the Caries Assessment Spectrum and Treatment (CAST) tool [23]. For complete information gathering to inform optimal individualised caries management, a structured clinical examination protocol is needed that has to include VE as the method of first choice (Fig. 2). When considering the requirements for an accurate and repeatable VE of teeth, the presence of other hard tissue defects, e.g., erosive tooth wear, tooth attrition, staining or developmental disorders such as molar-incisor hypomineralisation or enamel fluorosis, and the importance of caries activity and risk assessment must also be emphasised. The presence of biofilm requires documentation, and then it needs to be removed and the tooth surface dried and well illuminated prior to VE to distinguish caries from other tooth hard tissue diseases (differential diagnostics) and to document parameters relevant to the subsequent activity assessment. Once a caries lesion is detected, severity staging should involve the use of detailed and validated indices. For this, the ICDAS is probably the most widely used contemporary method [12–18]. For dental practitioners, the following categories might be of most clinical relevance [24, 25]:

- *Initial/ non-cavitated lesions.* The discolouration of initial lesions can vary and ranges from white-opaque, white‒brown to brown enamel demineralisation, depending on the age of the lesion and whether it is on occlusal or smooth surfaces. Early non-cavitated caries lesions can be detected better after drying of the tooth surface with pressured air for a few seconds (ICDAS score 1). Established lesions are recordable on wet surfaces (ICDAS score 2).
- *Moderate/ non-cavitated lesions.* A moderate lesion that shows either breakdown of the enamel surface (ICDAS score 3) or an underlying dentine shadow (ICDAS score 4) is probably associated with demineralisation into outer dentine.
- *Extensive/ cavitated lesions.* Distinct and extensive cavities (ICDAS score 5 and 6) show exposed dentine at its base and, if deeper, also along the lateral walls.

*Quality of the literature.* Several systematic reviews (SRs) have been published on the diagnostic accuracy of visual caries detection and diagnostic methods [18, 19, 26–29], and all of them included data for detecting primary caries mainly on occlusal and proximal surfaces in primary or permanent teeth. Only a minority of the included studies were conducted in a clinical setting [28, 30, 31], which may limit the generalizability of the documented findings. Furthermore, different sources of bias were detected and discussed [18, 29–32]. However, the most recently published SRs that were focused on the detection of enamel [29] and dentine lesions [30, 31] observed good or at least acceptable sensitivity and specificity values for VE. Visual inspection for detecting more advanced proximal lesions presented a low sensitivity, which is probably caused by the intact marginal ridge. The evidence for the clinical use of auxiliary tools such as magnification loupes is weak thus far [33, 34]. However, their practical use to aid VE should be considered, but decision making should be made with caution due to their potential to recommend unnecessary treatment [33, 34].

A shortcoming of VE is the difficulty of objectively recording the observations. Digital intraoral photographs may help to overcome this problem, especially in the case of lesions with a need for monitoring. Using photographs to assess occlusal surfaces with the ICDAS criteria was shown to be a suitable possibility for caries detection [35], but it is usually not part of everyday dental routines.

*Consensus recommendations for visual examination.* The expert group consented to the following key statements for VE:

1. Visual-tactile examination with structured classification systems is recommended as the method of first choice for the detection and staging of caries lesions on accessible surfaces. This requires appropriate illumination, cleaning after assessing biofilm presence and air-drying.
  Level of evidence: High.
  Level of agreement: 100% (agree, *N* = 20)/0% (neutral)/0% (disagree).
2. Visual-tactile examination should also be used to collect information on caries lesion activity.
  Level of evidence: Moderate.
  Level of agreement: 90% (agree, *N* = 18)/10% (neutral, *N* = 2)/0% (disagree).
3. Visual-tactile examination is also recommended in the context of differential diagnosis for all other dental hard tissue defects.
  Level of evidence: -.
  Level of agreement: 95% (agree, *N* = 19)/5% (neutral, *N* = 1)/0% (disagree).

### Dental radiography

One of the most frequent reasons for using dental radiography is to aid in caries detection. As dental caries involves demineralisation of enamel and dentine structure, sometimes leading to cavitation, the difference in X-ray attenuation between sound and carious tissue may allow an image of the lesion to be produced. Superimposition of sound enamel and dentine, however, means that lesions may be partially or completely hidden on radiographs, depending on the surface affected and the direction of the X-ray beam.

*Radiation protection aspects.* Dental radiography has been estimated to account for 13% of all diagnostic radiographical examinations worldwide and 32% of all plain radiographic procedures in Europe, with a conservative global estimation of at least 1.5 billion dental radiographic examinations annually [36]. Following the aim of protecting patients from potential hazards, the two fundamental principles of radiation protection that are relevant to patients – justification and optimisation – must be considered. Justification is the process of determining whether the use of the given radiographical procedure is expected to yield benefits to the individual who undergoes the procedure and to society that outweigh the harm (including radiation detriment) resulting from the procedure. The decision to use diagnostic radiographs must be justified individually for each patient based on consideration of the risk and the likely benefits. Children have the highest level of risk from X-ray exposure, approximately two to three times that of a 30-year-old patient, with risk becoming steadily less with advancing age. It is an absolute requirement of the justification process that the clinician should have taken a clinical history and performed an examination *before* selecting and performing radiographic imaging. As the radiation doses are generally low for dental radiography, the justification process pays particular attention to the anticipated benefits of the radiographic examination. In the context of the current document, a benefit can only arise if the clinician’s diagnostic accuracy is better when using the radiograph than it is without it.

Referral (or selection) criteria, produced by professional bodies, can assist the clinician in choosing the right kind of radiographic examination and the right time to perform it. Once justified, every effort must be taken to keep radiation doses as low as diagnostically acceptable (optimisation). This requires attention to the specific clinical indications and patient characteristics. Methods of dose optimisation have been described fully [36, 37] but include good patient co-operation, excellent operator techniques including appropriate choice of equipment and materials, correct setting of exposure parameters, limiting the field size to the minimum needed, ideal display and viewing of images, and a programme of quality assurance. Because ionising radiation is potentially hazardous to patients and clinical staff, there are legal constraints and requirements in performing radiographic examinations in dentistry, devised and enforced by the relevant government and/or health authority. These radiation protection requirements must be adhered to by clinicians [36].

*Imaging methods.* Caries lesions can be imaged using different dental radiographic methods, which have advantages and limitations (Table 2). In this context, intraoral bitewing, periapical, conventional panoramic radiographs and panoramic extraoral bitewings must be considered. Bitewing and periapical radiographs are the most widely used imaging techniques in dentistry. Both provide lateral projections of the teeth. Bitewing examinations are the standard radiographic method for caries detection in posterior teeth and should provide optimal geometric relationships between the ionising radiation beam, the teeth and the image receptor. Periapical radiographs are also sometimes used but may have unfavourable vertical beam angulations, particularly in the maxilla. Bitewing radiography can be challenging to perform in some patient groups, e.g., young children or patients with certain disabilities. Overlap of dental contact points is a common problem due to incorrect beam angulation, impacting the diagnostic accuracy for proximal caries.

**Table 2:** Overview of key findings for different radiographic techniques, including advantages, disadvantages and their usefulness in caries detection and diagnosis for the different sites at which caries might occur. Caries on the buccal and lingual surfaces of teeth are completely accessible to visual examination, so radiography would normally have no role to play. The table is based on both scientific evidence and expert opinion

Radiographic technique	Advantages	Disadvantages	Caries Diagnosis Applicability
Bitewing radiography	Correct geometry allows for: • Optimal geometry for proximal tooth surface assessment • Optimal geometry for alveolar bone level assessment • Maxillary and mandibular teeth captured in one image • High image resolution (high image detail) • Relatively low radiation dose (1–5 µSievert) • Low economic costs	• Not always comfortable for every patient • Not easy to position a holder if opposing teeth are missing (geometry may be compromised) • In case of dental crowding interproximal dental surfaces cannot always be visualised (overlapping projections) • More than one image sometimes needed per side in a fully dentate patient. • Only two-dimensional	**Occlusal caries** • No diagnostic value for enamel-only lesions • Moderate sensitivity and high specificity for dentine lesions • Has value for dentine lesions which, on visual examination, show initial enamel demineralisation. **Proximal caries** • Low to moderate sensitivity and high specificity for early lesions • low risk of false positive diagnoses • Higher sensitivity for deeper lesions **Root surface caries** • Low level of evidence available for diagnostic accuracy • Risk of misinterpreting “cervical burn-out” for caries and vice versa **Recurrent caries** • Moderate sensitivity with relatively high specificity, with greater value for proximal surfaces. • Risk of interpreting residual caries, deliberately left in deep cavities, as recurrent caries. • Risk of interpreting restoration deficiencies as recurrent caries.
Periapical radiography	Correct geometry allows for: • convenient interproximal tooth surface assessment • alveolar bone level assessment • relatively low radiation dose (1–5 µ Sievert) • Low economic costs	• Not always comfortable for every patient • might not have ideal vertical X-ray beam angulation for caries detection • Not easy to position a holder if opposing teeth are missing (geometry may be compromised) • In case of dental crowding, interproximal dental surfaces cannot always be visualised (overlapping contact points) • Multiple radiographs needed to image all the teeth in most patients • Only two-dimensional	Limited evidence on periapical radiography, as research is overwhelmingly on bitewings, but diagnostic accuracy is likely to be similar assuming paralleling technique is used. Any vertical angulation on the radiograph, relative to a bitewing, is likely to reduce diagnostic accuracy. **Occlusal caries** • As for bitewing radiography if image geometry is ideal **Proximal caries** • As for bitewing radiography if image geometry is ideal **Root surface caries** • As for bitewing radiography if image geometry is ideal **Recurrent caries** • As for bitewing radiography if image geometry is ideal • Vertical X-ray beam angulations might conceal lesions below restorations.
Panoramic radiography	• If patient is positioned according to manufacturer’s guidelines, a diagnostic overview of both maxilla and mandible is produced with minimal interproximal overlap between teeth • relatively low radiation dose (on average 24 µSievert) • Moderate economic costs	• If patient is not positioned according to manufacturer’s guidelines, an inferior quality overview of both maxilla and mandible is produced with often enormous interproximal overlap of teeth • Secondary “ghost” images may compromise interpretation • Lower image resolution than intraoral radiography • Good patient cooperation needed in view of extended exposure time • Lower image resolution than intraoral radiography • Only two-dimensional	No systematic review evidence. Most diagnostic accuracy research is old and lacks relevance to modern panoramic X-ray systems **Occlusal caries** • Has some diagnostic value for dentinal lesions **Proximal caries** • Lower sensitivity likely due to overlap of contact points and/or overlying soft tissue and air • Lower specificity possible due to overlying radiolucency from air in the oral cavity **Root surface caries** • As for proximal caries, although overlap of roots of adjacent teeth less likely. **Recurrent caries** • Likely to be similar to bitewing radiography, although diagnostic accuracy might be reduced by lower image resolution
Modified panoramic radiography – “extraoral bitewing”	• If patient is positioned according to manufacturer’s guidelines a periapical view of both maxillary and mandibular posterior teeth is produced with minimal interproximal overlap between teeth • Useful for patients who cannot tolerate intraoral radiography • relatively low radiation dose (approximately 15 µSievert) • Moderate economic costs	• If patient is not positioned according to manufacturer’s guidelines, an inferior quality overview of both maxilla and mandible is produced with often enormous interproximal overlap between teeth • Ghost images may compromise interpretation • Lower image resolution than intraoral radiography • Good patient cooperation needed in view of extended exposure time • Lower image resolution than intraoral radiography • Only two-dimensional	No systematic review evidence. Very limited primary research studies. **Occlusal caries** • As for conventional panoramic radiography. Deep lesions can be well demonstrated. **Proximal caries** • Assuming fewer overlapping contact points, sensitivity is similar to that with conventional bitewing radiography **Root surface caries** • As for conventional panoramic radiography **Recurrent caries** • As for conventional panoramic radiography
Cone beam computed tomography (CBCT)	• “Three dimensional” imaging of teeth is provided by multiplanar reformatting • Might assist in assessment of cavitation of proximal caries	• Streaking and beam hardening artefacts may compromise interpretation, leading to false positive diagnoses • Lower image resolution than intraoral or panoramic radiography • Motion artefacts common due to long scanning times radiation dose relatively high, varying with field of view, resolution and mA settings (10-1100 µSievert) • High economic costs	Current evidence and guidelines suggest CBCT should not normally be used specifically for caries diagnosis, but that scans taken for other reasons should be examined for dental caries. For all caries types, detection can be substantially reduced if artefact from metal (e.g. crowns, implants) is present **Occlusal caries** • Higher sensitivity than radiography in the absence of restorations but lower specificity **Proximal caries** • Higher sensitivity and similar specificity to radiography in the absence of restorations • Might be able to detect cavitation **Root surface caries** • No systematic review evidence for diagnostic accuracy • Most likely, better diagnostic accuracy than for radiography **Recurrent caries** • No systematic review evidence for diagnostic accuracy. • Likely reduction of sensitivity and specificity

Panoramic radiography also provides lateral-type images of the teeth, but does so in a different way to intraoral radiography, by means of a modified form of tomography. This can result in distortion of tooth shape and size according to position relative to the focal plane of the equipment and overlapping contact points, reducing the potential for proximal caries detection. It also provides lower image resolution than intraoral radiography. In recent years, several manufacturers have developed software applications to optimise the beam angulation so that overlap between proximal surfaces is reduced. This is sometimes associated with limitation of the field size to the posterior teeth to lower radiation dose. The terms “panoramic bitewing”, “extraoral bitewing” and others have been applied, but the term “panoramic extraoral bitewing” will be used in this document.

A few studies of diagnostic accuracy for proximal caries exist using panoramic extraoral bitewings, all but one of which have found no significant difference in ROC curve analysis between extraoral bitewing and conventional bitewing radiography [38–42], although one study reported a statistically significantly lower specificity and another lower ROC curve area for some observers using panoramic extraoral bitewings [38, 39]. While there is evidence that there is less proximal overlap of teeth compared with conventional panoramic radiographs, it is not eliminated [40]. It is important to emphasise that there are design differences in panoramic equipment from different manufacturers, which means that results from research studies might not apply generally.

Cone beam computed tomography (CBCT) is a cross-sectional X-ray imaging technique that has become more widely used in dentistry. Cross-sectional images can show caries lesions without superimposition of sound tooth tissues, but spatial resolution is inferior to both intraoral and panoramic radiography, and radiation doses are typically at least an order of magnitude greater. In clinical practice, artefacts from metallic or other high attenuation materials in the plane of the scan field of view can obliterate parts of the image or introduce dark areas that could mimic caries. Patient movement is more common with CBCT, reducing image quality. Different commercially available CBCT equipment varies in image quality, so what is true for one CBCT machine might not apply to others.

Recently, devices for computer-aided diagnosis or image methods of artificial intelligence (AI) have been introduced to the market for several medical indications, including those in dentistry [42, 43]. They are based on the automated processing of image datasets using special AI-powered algorithms. These systems must be properly trained with an adequate number of suitable images/diagnostic datasets. To date, experience in the field of caries detection and diagnosis is limited, and quality standards are warranted [44–46]. At the current time, it is too soon to provide evidence-based guidance on using AI methods to assist in caries detection, except to emphasise that their role is likely to remain as a help to clinicians, who remain responsible for the final diagnosis.

*Quality of the literature.* All recently published SRs were concerned with diagnostic accuracy using intraoral radiography for caries. The focus of these SRs varied according to the eligibility criteria used, including factors such as the type of caries (tooth site, lesion depth), the dentition and the types of primary study included (laboratory and/or clinical). Most addressed proximal or occlusal caries. None of the SRs that were analysed considered buccal surface or lingual/palatal surface dental decay, but it can be assumed that VE should suffice to diagnose caries on these surfaces. A recent series of SRs addressing the uses of intraoral radiography [47], panoramic radiography [48], CBCT [49] and radiation doses in dental radiography [37] within the paediatric age group, along with a linked overview paper [50], were also considered. No SR exists of studies specifically assessing diagnostic accuracy using conventional panoramic radiography or panoramic extraoral bitewing radiography for dental caries. One SR focused on early dental caries, including studies using CBCT [51], was available. A further contemporary narrative review paper on the radiological diagnosis of caries was also used [52]. From the SRs we analysed, it was apparent that there is often a high risk of bias in studies, which jeopardises a correct and relevant interpretation of the data on the use of radiographs in the diagnosis of caries. Laboratory (in vitro) primary studies were more frequent than clinical (in vivo) primary studies of diagnostic accuracy [30, 31]. Both designs have strengths and limitations, but in vitro research has advantages in the consistency of methods and the possibility of a reference standard that is reproducible, that represents the ground truth and is fully independent of the radiographical technique being investigated. However, laboratory studies would have limited external validity since the images are produced with higher quality and less interference from overlapping anatomical structures. The quality of the SRs themselves was not assessed objectively by us, and there was no “umbrella” review (meta-review) of SRs to assess their risk of bias. From the SR evidence, some general findings were identified in relation to using intraoral (bitewing) radiography for caries detection and diagnosis:

- Intraoral radiography is a useful diagnostic test, more so for proximal caries than for occlusal caries and with greater value for dentine and cavitated lesions [53].
- Intraoral radiography typically gives low to moderate sensitivity (approximately ∼ 0.40–0.60) but high specificity (∼ 0.80–0.90); sensitivity was mostly higher for proximal caries and more progressed/ dentin caries lesions in comparison to occlusal surfaces and early caries stages [30, 31, 51, 53, 54]. Therefore, for detection of early lesions intraoral (bitewing) radiography and visual examination should be used together. Here, VE can access easily occlusal surfaces, but has limitations on proximal sites which indicate the use intraoral radiography.
- Sensitivity tends to be lower for early caries and higher for more advanced caries.
- Broadly similar findings apply to primary and permanent dentition and to analogue and digital forms of radiography [30].

For panoramic radiography, in the absence of SR evidence, two general points are made based on the experience of the authors and their knowledge of primary research studies:

- There is some evidence that panoramic extraoral bitewings can provide similar levels of diagnostic accuracy as intraoral (bitewing) radiography, so long as images are technically excellent.
- Unavoidable overlap of proximal surfaces, intrinsic to panoramic radiography, remains a common problem that can reduce diagnostic accuracy for caries.

For CBCT, the limited SR evidence yielded three general findings:

- Summary values for sensitivity and specificity for proximal caries were 0.68 and 0.90, respectively [51].
- For occlusal caries, the sensitivity was also higher than that for intraoral radiography (0.76), but the specificity was lower (0.54), which would mean an unacceptable false positive rate.
- There is some evidence that cavitation of proximal caries lesions can be detected effectively using CBCT [49].

*Consensus recommendations for dental radiography.* Every radiographic examination should be clinically justified on a patient-individual basis, in accordance with basic principles in radiation protection and local legal requirements (principle of justification). Optimal diagnostic accuracy requires correct exposure parameters, good patient cooperation, excellent operator techniques and ideal viewing conditions for interpretation (principle of optimisation). The following recommendations were agreed upon:

4. Intraoral – preferably bitewing – radiography is recommended as an additional method to VE for caries lesion detection and staging.
  Intraoral – preferably bitewing radiography is recommended as an additional method to VE for caries lesion detection and staging.
  Level of evidence: Moderate.
  Level of agreement: 100% (agree, *N* = 20)/0% (neutral)/0% (disagree).
5. Extraoral bitewing radiography by panoramic equipment can be considered only if conventional bitewing radiographs are not feasible to perform.
  Level of evidence: Low.
  Level of agreement: 80% (agree, *N* = 16)/20% (neutral, *N* = 4)/0% (disagree).
6. Other radiographical images, e.g., conventional panoramic radiography or CBCT, should not be taken for the sole purpose of caries lesion detection and staging, but those taken for other purposes should be inspected for incidental findings of caries.
  Level of evidence: -.
  Level of agreement: 90% (agree, *N* = 18)/10% (neutral, *N* = 2)/0% (disagree).

### Non-radiographical caries diagnostic methods

While dental radiography – especially bitewing radiographs – provides valid, reproducible and clinically relevant information, it must be stated again that an unrestricted use of ionising radiation is unacceptable due to its undesired biological effects. This justifies the general need for adjunct, ionising radiation-free caries detection and diagnostic methods. The available – mostly optical – methods can be systematised by the excitation wavelength:

- *Red fluorescence*: DIAGNOdent 2095 and 2190 (KaVo, Biberach, Germany; excitation wavelength of 655 nm; DIAGNOdent 2095 is out of production), Midwest Caries ID (Dentsply, York, Pennsylvania; excitation wavelength of ∼ 650 nm, out of production) and the Canary System (Quantum Dental Technologies Inc., Toronto, Ontario, Canada).
- *Green & blue fluorescence*: VistaCam iX intraoral camera (Dürr Dental SE, Bietigheim-Bissingen, Germany, excitation wavelength of ∼ 405 nm), VistaProof intraoral camera (Dürr Dental SE, Bietigheim-Bissingen, Germany, excitation wavelength of ∼ 405 nm, out of production), CamX Spectra Caries Detection Aid (Air Techniques, Melville, NY, USA), Soprolife camera (Acteon, La Ciotat, France; blue excitation wavelength of ∼ 420 nm) and quantitative light-induced fluorescence cameras (QLFD, QRAYcam Pro, QRAYpen and QRAYscan, Inspektor Research Systems BV, Amsterdam, The Netherlands; violet‒blue excitation wavelength of ∼ 405 nm and detects green (∼ 520 nm) and red fluorescence (600–670 nm)).
- *(Near)Infrared light*: DIAGNOcam/CariVu intraoral camera (KaVo, Biberach, Germany; transilluminating wavelength of 780 nm), VistaCam intraoral camera (Dürr Dental SE, Bietigheim-Bissingen, Germany, excitation wavelength = 850 nm) and iTero Element 5 intraoral scanner (Align Technology Switzerland, Rotkreuz, Switzerland, excitation wavelength = 850 nm).
- *Others*: electrical resistance measurements (e.g., ECM, Lode Diagnostics, Groningen, The Netherlands, out of production) and AC impedance spectroscopy technique (ACIST, CarieScan Pro, CarieScan, Charlotte, NC, USA).

As mentioned before, several devices with different, mostly light-based, technologies have been introduced or will appear periodically to the dental market. Some of them are now out of production and, therefore, not available anymore. From the dental practitioner´s point of view, adjunct diagnostic methods should be usable on all tooth surfaces in both dentitions, have a good diagnostic performance in terms of accuracy and reproducibility, especially on proximal and occlusal surfaces, be easy to use and offer an imaging feature that objectively captures changes over time in relation to relevant anatomical structures, e.g., dental pulp, instead of simple categorial or numeric measures. It is important to recognise that bitewing radiography fulfils most of these requirements but has not been fully reached by any of the available adjunct diagnostic methods thus far. This aspect – and the acquisition costs – must be understood as the main reasons for its limited attention and use in dental practices. However, with respect to the need for ionising radiation, the clinical use of bitewing radiographs can potentially be lowered nowadays and should ideally be phased out in the future, if and when equivalent adjunct technologies become available. This should motivate researchers and manufacturers to improve existing devices or to develop new ionising radiation-free technologies for caries detection (Fig. 2).

*Quality of the literature.* When summarising the existing evidence for those adjunct methods that have reached some attention in dental practices, the available SRs and meta-analyses should be preferably considered in this context [30, 31, 54–60]. The indication for using adjunct diagnostic methods has to be seen, especially in patients with increased caries activity and risk, as well as to investigate non-accessible proximal surfaces or to perform an assessment including staging, classification and monitoring on early, non-cavitated caries lesions [61]. The detection of caries-related cavities is of little significance, as such defects can be detected by VE.

Laser-induced fluorescence-based measurements with DIAGNOdent 2095 and 2190 gained popularity for the evaluation of occlusal and proximal surfaces in posterior teeth just after the turn of the millennium. Both devices have been scientifically validated in many clinical studies, and from today´s perspective, they are the most extensively proven adjunct diagnostic method for caries detection. The diagnostic performance in terms of accuracy was mostly assessed as good to acceptable on proximal surfaces and occlusal surfaces [30, 31, 54, 62]. Gimenez et al. [60] observed better accuracy in detecting more advanced caries lesions. Importantly, accuracy depends on the clinical covariables, e.g., tooth cleaning and drying, presence of staining, etc., which may result clinically in higher fluorescence values and an increased likelihood of false-positive diagnoses [54]. Therefore, higher readings need to be interpreted with caution, and additional imaging methods might be indicated to verify unclear findings at specific thresholds to avoid false-positive diagnoses [63].

Approximately a decade after the introduction of fluorescence-based measurements, two caries imaging technologies were successfully released on the dental market and gained interest from practitioners: photo-optical, fluorescence-based intraoral cameras (VistaProof/VistaCam) and near-infrared-based transillumination devices (DIAGNOcam/CariVu and VistaCam). Both manufacturers merged their fluorescence- and near-infrared-based technologies later separately into 3-in-1 intraoral camera systems to increase the diagnostic value for the dentist. When considering the SR by Ortiz et al. [58], the authors reported good overall accuracy for the detection of interproximal primary caries with near-infrared light transillumination and argued that the method could be routinely used for dental check-ups, especially in high-risk caries populations and in patients where the use of radiation should be reduced, such as for pregnant women or children [58]. Interestingly, Macey et al. [56] came to the opposite conclusion and referred to the limited ability of near-infrared transillumination to detect enamel caries.

*Consensus recommendations for adjunct diagnostic methods.* With respect to the number of available devices, the need to evaluate each method in the primary and permanent dentition on different tooth surfaces/sites, e.g., occlusal, proximal, smooth or root surfaces, with diverging caries detection thresholds, e.g., caries detection, dentine caries detection or cavitation detection [32], it needs to be highlighted that the available clinical studies provide only incomplete data in comparison to the overall information needed [55–57]. In addition, there was a consistently reported lack of clinical studies, heterogeneity between trials and relevant risk of bias in several study reports, which potentially limits the generalizability of published data [32, 64]. Taking these limitations into account, the following evidence-based recommendations can be provided:

7. Adjunct, non-ionising radiation and methods for caries lesion detection and/or staging - especially on non-accessible surfaces - may be preferred if radiography is not feasible or considered due to the individual clinical situation.
  Level of evidence: Moderate.
  Level of agreement: 85% (agree, *N* = 17)/15% (neutral, *N* = 3)/0% (disagree).
8. Photography or intraoral scans may be used to record and compare the caries status over time.
  Level of evidence: -.
  Level of agreement: 85% (agree, *N* = 17)/15% (neutral, *N* = 3)/0% (disagree).

### Knowledge gaps

A major limitation identified was the lack of clinical studies on caries detection methods [18, 27, 28, 30, 31, 60] in comparison to the frequent availability of laboratory trials. This resulted in the fact that the quality of evidence was found to be moderate or low for most of the given recommendations. The importance of the clinical setting also needs to be highlighted from the dental practitioner´s perspective. In terms of applicability, future studies should be conducted in a clinical setting that is representative of the complexities encountered in primary and secondary caries assessment [65]. Longitudinal clinical studies monitoring caries lesions with different methods over time and measuring variables closely linked with caries progression or stagnation should be further conducted to evaluate the benefits and harms of diagnostic strategies for patients and health care systems [29]. Furthermore, the existing methodological heterogeneity [32] has to be mentioned as another aspect that potentially limits unbiased comparisons between studies. Therefore, future investigations should minimise potential sources of bias, e.g., selection/spectrum, verification, validation or reproducibility bias, as best as possible, aiming at enabling clear and comprehensive study reporting [29, 31]. Another issue which needs to be addressed in future diagnostic studies are changing threshold values which are linked with new or modified caries management strategies. This indicates the need to proof new treatment concepts also from the diagnostic point of view aiming at avoiding false negatives and false positive determinations.

A further knowledge gap related to the recently introduced AI-based methods for automated analysis of dental radiographs or intraoral photographs has been identified [45, 46, 52, 66, 67], for which no applied studies are available thus far. Research on AI is also rapidly developing in dentistry [68], with dental caries detection being one main area of interest [43]. Challenges may exist in developing AI methods, including building large datasets, developing generalizable models and validating such methods against representative benchmarked datasets by using useful metrics and outcomes. Currently, it is too soon to provide evidence-based guidance on using AI-based methods to assist in caries detection. Furthermore, it seems likely that 3D scanners will be equipped with caries detection features, which results in the foreseeable future that such devices will become multiusable.

## Conclusions

The expert panel merged existing evidence from the scientific literature with practical considerations and provided recommendations for their use in daily dental clinical practice. Here, visual examination with structured classification systems is recommended as the method of first choice for the detection and assessment of caries lesions on accessible surfaces. Intraoral – preferably bitewing – radiography is recommended as an additional method for caries detection. Adjunct, non-ionizing radiation and methods for caries lesion detection and/or assessment might also be useful in indicated clinical situations.

## Data Availability

No datasets were generated or analysed during the current study.

## References

[1] Neuhaus KW, Kühnisch J, Banerjee A, Martignon S, Ricketts D, Schwendicke F, van der Veen MH, Doméjean S, Fontana M, Lussi A, Jablonski-Momeni A, Mendes FM, Douglas GVA, Schmalz G, Campus G, Aps J, Horner K, Opdam N, Huysmans MCDNJM, Splieth C (2024) : How to assess caries lesion activity and caries progression? A joint ORCA and EFCD expert Delphi consensus statement. Caries Res submitted

[2] Huysmans MCDNJM, Fontana M, Lussi A, Jablonski-Momeni A, Banerjee A, Ricketts D, Schwendicke F, Mendes FM, Douglas G, Schmalz G, Campus G, Aps JKM, Horner K, Neuhaus K, van der Veen M, Opdam N, Domejean S, Martignon S, Kühnisch J, Splieth C (2024) : Recommendations on caries diagnosis at the individual level. An ORCA/EFCD consensus document. Caries Res submitted

[3] Machiulskiene V, Campus G, Carvalho JC, Dige I, Ekstrand KR, Jablonski-Momeni A, Maltz M, Manton DJ, Martignon S, Martinez-Mier EA, Pitts NB, Schulte AG, Splieth CH, Tenuta LMA, Ferreira Zandona A, Nyvad B (2020). Terminology of Dental Caries and Dental Caries Management: Consensus Report of a Workshop Organized by ORCA and Cariology Research Group of IADR. Caries Res.

[4] Paris S, Banerjee A, Bottenberg P, Breschi L, Campus G, Doméjean S, Ekstrand K, Giacaman RA, Haak R, Hannig M, Hickel R, Juric H, Lussi A, Machiulskiene V, Manton D, Jablonski-Momeni A, Santamaria R, Schwendicke F, Splieth CH, Tassery H, Zandona A, Zero D, Zimmer S, Opdam N (2020). How to intervene in the Caries process in older adults: a joint ORCA and EFCD Expert Delphi Consensus Statement. Caries Res.

[5] Santamaría RM, Abudrya MH, Gül G, Mourad MS, Gomez GF, Zandona AGF (2020). How to intervene in the Caries process: Dentin Caries in primary teeth. Caries Res.

[6] Schwendicke F, Splieth CH, Bottenberg P, Breschi L, Campus G, Doméjean S, Ekstrand K, Giacaman RA, Haak R, Hannig M, Hickel R, Juric H, Lussi A, Machiulskiene V, Manton D, Jablonski-Momeni A, Opdam N, Paris S, Santamaria R, Tassery H, Zandona A, Zero D, Zimmer S, Banerjee A (2020). How to intervene in the caries process in adults: proximal and secondary caries? An EFCD-ORCA-DGZ expert Delphi consensus statement. Clin Oral Investig.

[7] Splieth CH, Banerjee A, Bottenberg P, Breschi L, Campus G, Ekstrand KR, Giacaman RA, Haak R, Hannig M, Hickel R, Juric H, Lussi A, Machiulskiene V, Manton DJ, Jablonski-Momeni A, Opdam NJM, Paris S, Santamaría RM, Schwendicke F, Tassery H, Ferreira Zandona A, Zero DT, Zimmer S, Doméjean S (2020). How to intervene in the Caries process in children: a joint ORCA and EFCD Expert Delphi Consensus Statement. Caries Res.

[8] Askar H, Krois J, Göstemeyer G, Bottenberg P, Zero D, Banerjee A, Schwendicke F (2020). Secondary caries: what is it, and how it can be controlled, detected, and managed?. Clin Oral Investig.

[9] Splieth CH, Kanzow P, Wiegand A, Schmoeckel J, Jablonski-Momeni A (2020). How to intervene in the caries process: proximal caries in adolescents and adults-a systematic review and meta-analysis. Clin Oral Investig.

[10] Meyer-Lueckel H, Machiulskiene V, Giacaman RA (2019). How to intervene in the Root caries process? Systematic review and Meta-analyses. Caries Res.

[11] Moher D, Schulz KF, Simera I, Altman DG (2010). Guidance for developers of health research reporting guidelines. PLoS Med.

[12] Ismail AI, Sohn W, Tellez M, Amaya A, Sen A, Hasson H, Pitts NB (2007). The International Caries Detection and Assessment System (ICDAS): an integrated system for measuring dental caries. Community Dent Oral Epidemiol.

[13] Pitts N (ed) (2009) Detection

[14] Ekstrand KR, Gimenez T, Ferreira FR, Mendes FM, Braga MM (2018). The International Caries Detection and Assessment System - ICDAS: a systematic review. Caries Res.

[15] ElSalhy M, Ali U, Lai H, Flores-Mir C, Amin M (2019). Caries reporting in studies that used the International Caries Detection and Assessment System: a scoping review. Community Dent Oral Epidemiol.

[16] Foros P, Oikonomou E, Koletsi D, Rahiotis C (2021). Detection methods for early caries diagnosis: a systematic review and Meta-analysis. Caries Res.

[17] Gomez J, Tellez M, Pretty IA, Ellwood RP, Ismail AI (2013). Non-cavitated carious lesions detection methods: a systematic review. Community Dent Oral Epidemiol.

[18] Gimenez T, Piovesan C, Braga MM, Raggio DP, Deery C, Ricketts DN, Ekstrand KR, Mendes FM (2015). Visual inspection for Caries Detection: a systematic review and Meta-analysis. J Dent Res.

[19] Ekstrand KR, Ricketts DN, Kidd EA (1997). Reproducibility and accuracy of three methods for assessment of demineralization depth of the occlusal surface: an in vitro examination. Caries Res.

[20] Nyvad B, Machiulskiene V, Baelum V (1999). Reliability of a new caries diagnostic system differentiating between active and inactive caries lesions. Caries Res.

[21] Kühnisch J, Goddon I, Berger S, Senkel H, Bücher K, Oehme T, Hickel R, Heinrich-Weltzien R (2009). Development, Methodology and potential of the New Universal Visual Scoring System (UniViSS) for Caries Detection and diagnosis. Int J Environ Res Public Health.

[22] Kühnisch J, Bücher K, Henschel V, Albrecht A, Garcia-Godoy F, Mansmann U, Hickel R, Heinrich-Weltzien R (2011). Diagnostic performance of the Universal Visual Scoring System (UniViSS) on occlusal surfaces. Clin Oral Investig.

[23] Frencken JE, de Amorim RG, Faber J, Leal SC (2011). The Caries Assessment Spectrum and Treatment (CAST) index: rational and development. Int Dent J.

[24] Martignon S, Pitts NB, Goffin G, Mazevet M, Douglas GVA, Newton JT, Twetman S, Deery C, Doméjean S, Jablonski-Momeni A, Banerjee A, Kolker J, Ricketts D, Santamaria RM (2019). CariesCare practice guide: consensus on evidence into practice. Br Dent J.

[25] Pitts NB, Banerjee A, Mazevet ME, Goffin G, Martignon S (2021). From ‘ICDAS’ to ‘CariesCare International’: the 20-year journey building international consensus to take caries evidence into clinical practice. Br Dent J.

[26] Bader JD, Shugars DA, Bonito AJ (2002). A systematic review of the performance of methods for identifying carious lesions. J Public Health Dent.

[27] Gimenez T, Piovesan C, Braga MM, Raggio DP, Deery C, Ricketts DN, Ekstrand KR, Mendes FM (2015). Clinical relevance of studies on the accuracy of visual inspection for detecting caries lesions: a systematic review. Caries Res.

[28] Gimenez T, Tedesco TK, Janoian F, Braga MM, Raggio DP, Deery C, Ricketts DNJ, Ekstrand KR, Mendes FM (2021). What is the most accurate method for detecting caries lesions? A systematic review. Community Dent Oral Epidemiol.

[29] Macey R, Walsh T, Riley P, Glenny AM, Worthington HV, O’Malley L, Clarkson JE, Ricketts D (2021). Visual or visual-tactile examination to detect and inform the diagnosis of enamel caries. Cochrane Database Syst Rev.

[30] Kapor S, Rankovic MJ, Khazaei Y, Crispin A, Schüler I, Krause F, Lussi A, Neuhaus K, Eggmann F, Michou S, Ekstrand K, Huysmans MC, Kühnisch J (2021). Systematic review and meta-analysis of diagnostic methods for occlusal surface caries. Clin Oral Investig.

[31] Janjic Rankovic M, Kapor S, Khazaei Y, Crispin A, Schüler I, Krause F, Ekstrand K, Michou S, Eggmann F, Lussi A, Huysmans M-C, Neuhaus K, Kühnisch J (2021). Systematic review and meta-analysis of caries diagnostic studies on proximal surfaces. Clin Oral Investig.

[32] Kühnisch J, Janjic Rankovic M, Kapor S, Schüler I, Krause F, Michou S, Ekstrand K, Eggmann F, Lussi A, Neuhaus K, Huysmans MC (2021). Identifying and avoiding risk of bias in caries diagnostic studies. J Clin Med.

[33] Goel D, Sandhu M, Jhingan P, Sachdev V (2016). Effectiveness of Air Drying and Magnification methods for detecting initial caries on Occlusal surfaces using three different diagnostic aids. J Clin Pediatr Dent.

[34] Neuhaus KW, Jost F, Perrin P, Lussi A (2015). Impact of different magnification levels on visual caries detection with ICDAS. J Dent.

[35] Bottenberg P, Jacquet W, Behrens C, Stachniss V, Jablonski-Momeni A (2016). Comparison of occlusal caries detection using the ICDAS criteria on extracted teeth or their photographs. BMC Oral Health.

[36] International Atomic Energy Agency, Radiation Protection in Dental Radiology (2022) Safety Report Series No. 108, IAEA, Vienna. https://www-pub.iaea.org/MTCD/Publications/PDF/PUB1972_Web.pdf

[37] Van Acker JWG, Pauwels NS, Cauwels RGEC, Rajasekharan S (2020). Outcomes of different radioprotective precautions in children undergoing dental radiography: a systematic review. Eur Arch Paediatr Dent.

[38] Gaalaas L, Tyndall D, Mol A, Everett ET, Bangdiwala A (2016). Ex vivo evaluation of new 2D and 3D dental radiographic technology for detecting caries. Dentomaxillofac Radiol.

[39] Kamburoglu K, Kolsuz E, Murat S, Yüksel S, Ozen T (2012). Proximal caries detection accuracy using intraoral bitewing radiography, extraoral bitewing radiography and panoramic radiography. Dentomaxillofac Radiol.

[40] Terry GL, Noujeim M, Langlais RP, Moore WS, Prihoda TJ (2016). A clinical comparison of extraoral panoramic and intraoral radiographic modalities for detecting proximal caries and visualizing open posterior interproximal contacts. Dentomaxillofac Radiol.

[41] Abu El-Ela WH, Farid MM, Mostafa MS (2016). Intraoral versus extraoral bitewing radiography in detection of enamel proximal caries: an ex vivo study. Dentomaxillofac Radiol.

[42] Abdinian M, Razavi SM, Faghihian R, Samety AA, Faghihian E (2015). Accuracy of Digital Bitewing Radiography versus different views of Digital Panoramic Radiography for Detection of Proximal Caries. J Dent (Tehran).

[43] Putra RH, Doi C, Yoda N, Astuti ER, Sasaki K (2022). Current applications and development of artificial intelligence for digital dental radiography. Dentomaxillofac Radiol.

[44] Schmalz G, Jakubovics N, Schwendicke F (2022). Normative approaches for oral health: Standards, specifications, and guidelines. J Dent Res.

[45] Schwendicke F, Cejudo Grano de Oro J, Garcia Cantu A, Meyer-Lueckel H, Chaurasia A, Krois J (2022). Artificial Intelligence for Caries Detection: value of data and information. J Dent Res.

[46] Schwendicke F, Büttner M (2023). Artificial intelligence: advances and pitfalls. Br Dent J.

[47] Aps JKM, Lim LZ, Tong HJ, Kalia B, Chou AM (2020). Diagnostic efficacy of and indications for intraoral radiographs in pediatric dentistry: a systematic review. Eur Arch Paediatr Dent.

[48] Tsiklakis K, Mitsea A, Tsichlaki A, Pandis N (2020). A systematic review of relative indications and contra-indications for prescribing panoramic radiographs in dental paediatric patients. Eur Arch Paediatr Dent.

[49] Horner K, Barry S, Dave M, Dixon C, Littlewood A, Pang CL, Sengupta A, Srinivasan V (2020). Diagnostic efficacy of cone beam computed tomography in paediatric dentistry: a systematic review. Eur Arch Paediatr Dent.

[50] Kühnisch J, Anttonen V, Duggal MS, Spyridonos ML, Rajasekharan S, Sobczak M, Stratigaki E, Van Acker JWG, Aps JKM, Horner K, Tsiklakis K (2020). Best clinical practice guidance for prescribing dental radiographs in children and adolescents: an EAPD policy document. Eur Arch Paediatr Dent.

[51] Walsh T, Macey R, Riley P, Glenny AM, Schwendicke F, Worthington HV, Clarkson JE, Ricketts D, Su TL, Sengupta A (2021). Imaging modalities to inform the detection and diagnosis of early caries. Cochrane Database Syst Rev.

[52] Wenzel A (2021). Radiographic modalities for diagnosis of caries in a historical perspective: from film to machine-intelligence supported systems. Dentomaxillofacial Radiol.

[53] Schwendicke F, Tzschoppe M, Paris S (2015). Radiographic caries detection: a systematic review and meta-analysis. J Dent.

[54] Bader JD, Shugars DA (2004). A systematic review of the performance of a laser fluorescence device for detecting caries. J Am Dent Assoc.

[55] Macey R, Walsh T, Riley P, Glenny AM, Worthington HV, Fee PA, Clarkson JE, Ricketts D (2020). Fluorescence devices for the detection of dental caries. Cochrane Database Syst Rev.

[56] Macey R, Walsh T, Riley P, Hogan R, Glenny AM, Worthington HV, Clarkson JE, Ricketts D (2021). Transillumination and optical coherence tomography for the detection and diagnosis of enamel caries. Cochrane Database Syst Rev.

[57] Macey R, Walsh T, Riley P, Glenny AM, Worthington HV, Clarkson JE, Ricketts D (2021). Electrical conductance for the detection of dental caries. Cochrane Database Syst Rev.

[58] Ortiz MIG, de Melo Alencar C, De Paula BLF, Magno MB, Maia LC, Silva CM (2020). Accuracy of near-infrared light transillumination (NILT) compared to bitewing radiograph for detection of interproximal caries in the permanent dentition: a systematic review and meta-analysis. J Dent.

[59] Doméjean S, Rongier J, Muller-Bolla M (2016). Detection of Occlusal Carious Lesion using the SoproLife Camera: a systematic review. J Contemp Dent Pract.

[60] Gimenez T, Braga MM, Raggio DP, Deery C, Ricketts DN, Mendes FM (2013). Fluorescence-based methods for detecting caries lesions: systematic review, meta-analysis and sources of heterogeneity. PLoS ONE.

[61] Gomez J (2015). Detection and diagnosis of the early caries lesion. BMC Oral Health.

[62] Twetman S (2015). Visual inspection Displays Good Accuracy for detecting caries lesions. J Evid Based Dent Pract.

[63] Schwendicke F, Brouwer F, Paris S, Stolpe M (2016). Detecting Proximal secondary caries lesions: a cost-effectiveness analysis. J Dent Res.

[64] Neuhaus K, Eggmann F, Kühnisch J, Kapor S, Janjic Rankovic M, Schüler I, Krause F, Lussi A, Ekstrand K, Michou S, Huysmans MC (2022). STAndard Reporting of CAries Detection and Diagnostic studies (STARCARDDS). Clin Oral Investig.

[65] Signori C, Gimenez T, Mendes FM, Huysmans MDNJM, Opdam NJM, Cenci MS (2018). Clinical relevance of studies on the visual and radiographic methods for detecting secondary caries lesions - a systematic review. J Dent.

[66] Prados-Privado M, García Villalón J, Martínez-Martínez CH, Ivorra C, Prados-Frutos JC (2020). Dental Caries diagnosis and detection using neural networks: a systematic review. J Clin Med.

[67] Moharrami M, Farmer J, Singhal S, Watson E, Glogauer M, Johnson AEW, Schwendicke F, Quinonez C (2023). Detecting dental caries on oral photographs using artificial intelligence: a systematic review. Oral Dis.

[68] Heo MS, Kim JE, Hwang JJ, Han SS, Kim JS, Yi WJ, Park IW (2021). Artificial intelligence in oral and maxillofacial radiology: what is currently possible?. Dentomaxillofac Radiol.

